# Effectiveness of Proprioceptive Neuromuscular Facilitation Compared with Standardized Exercise-Based Physiotherapy for Chronic Shoulder Pain Treated with Ultrasound-Guided Corticosteroid Injections: A Randomized Controlled Trial

**DOI:** 10.3390/jfmk11020224

**Published:** 2026-05-31

**Authors:** Alessandro de Sire, Andrea Demeco, Emanuele Prestifilippo, Rita Ilaria De Socio, Marco Mazzei, Annunziata Filippo, Stefano Fasano, Kristian Efremov, Nicola Marotta, Antonio Ammendolia

**Affiliations:** 1 Physical and Rehabilitative Medicine, Department of Medical and Surgical Sciences, University of Catanzaro “Magna Graecia”, 88100 Catanzaro, Italyammendolia@unicz.it (A.A.); 2 Research Center on Musculoskeletal Health, MusculoSkeletalHealth@UMG, University of Catanzaro “Magna Graecia”, 88100 Catanzaro, Italy; 3 Department of Orthopaedic Surgery, NYU-Langone Winthrop Hospital, Mineola, NY 11501, USA; 4 Physical and Rehabilitative Medicine, Department of Experimental and Clinical Medicine, University of Catanzaro “Magna Graecia”, 88100 Catanzaro, Italy

**Keywords:** chronic shoulder pain, subacromial bursitis, ultrasound-guided corticosteroid injections, PNF, standardized exercise-based physiotherapy, musculoskeletal pain, shoulder rehabilitation

## Abstract

**Background**: Chronic shoulder pain associated with subacromial bursitis is a common clinical condition characterized by pain and functional limitation, factors that contribute significantly to chronic morbidity for the patient. The combination of ultrasound-guided corticosteroid injections and physical therapy may improve clinical outcomes. This study compared the effectiveness of Proprioceptive Neuromuscular Facilitation and standardized exercise-based physiotherapy after a common protocol of ultrasound-guided corticosteroid injections in patients with chronic shoulder pain associated with subacromial bursitis. **Methods**: A randomized controlled pilot study was conducted on adult patients with chronic shoulder pain (NRS ≥ 4), who received 3 weekly intra-bursal ultrasound-guided injections of corticosteroids and local anesthetic, followed by either 10 sessions of PNF or standardized exercise-based physiotherapy. The primary outcome was pain intensity assessed by NRS. Secondary outcomes included DASH, EQ-5D, EQ-VAS, and ROM, assessed at baseline, 2 weeks, 4 weeks, 12 weeks, and 24 weeks. **Results**: The PNF group showed greater improvements in selected outcomes and at some follow-up time points, particularly for functional measures and shoulder ROM. However, between-group differences were not consistent across all predefined outcomes. **Conclusions**: Both PNF-based rehabilitation and standardized exercise-based physiotherapy may improve clinical outcomes after ultrasound-guided corticosteroid injection in patients with subacromial bursitis. However, the added value of PNF appears limited to selected outcomes and time points, and its superiority over standardized physiotherapy cannot be definitively established.

## 1. Introduction

Chronic shoulder pain is generally defined as pain and functional deficit that persists for longer than six months. This condition represents a significant burden on healthcare systems and is one of the main reasons for medical consultation within Orthopedics and Rehabilitation Medicine. It accounts for approximately 16% of all diagnosed musculoskeletal disorders [1]. The prevalence of shoulder disorders ranges from 7% to 34%, with subacromial impingement identified as one of the most common etiologies [2]. Other frequent conditions contributing to chronic shoulder pain include rotator cuff tendinopathies, glenohumeral osteoarthritis, and disorders of the long head of the biceps tendon. The high prevalence of subacromial impingement is linked to both intrinsic biomechanical factors of the scapulohumeral joint and repetitive functional overloads, often associated with occupational activities that require sustained overhead use of the upper extremity (e.g., carpenters, painters, hairdressers), as well as with specific sports disciplines, like volleyball and tennis [3]. Moreover, alterations in scapular kinematics, referred to as dyskinesia, have been shown to increase the risk of chronic shoulder pain, particularly in athletes and makes necessary a differential diagnosis [4,5,6].

Shoulder pain often follows a chronic and/or relapsing course, with approximately 54% of patients experiencing persistent symptoms after three years; such pain negatively affects motor function, psychophysical health, and personal autonomy, often impairing daily activities that require upper limb use [4,5,6].

Physiotherapy, with targeted therapeutic programs focusing on a limited number of specific exercises, can improve scapulohumeral coordination and optimize humeral head alignment [7]. However, current evidence remains limited and does not provide definite guidance for treatment options particularly in terms of robotics [8].

In this context, PNF may represent a valuable therapeutic approach. This manual rehabilitation technique uses diagonal and spiral movement patterns that integrate resistance and stretching of synergistic muscle groups throughout the full range of motion, respecting the anatomical orientation of the muscles from their origin to their insertion [9,10]. Evidence supports the use of PNF in the management of adhesive capsulitis, demonstrating significant benefits in both pain reduction and functional recovery [11,12]. PNF also enhances muscle flexibility and improves both active and passive range of motion [13]. Furthermore, it has also been applied in the rehabilitation of soft tissues to restore functional range of motion (ROM), muscle strength, coordination, and balance [14].

Infiltrative therapy is generally considered when conservative strategies such as physiotherapy or systemic medications are ineffective. It consists of the administration of medication via intra-articular, periarticular, intramuscular, or perineural injections for analgesic, anti-inflammatory, antispastic, or diagnostic purposes [15]. In the management of shoulder disorders, anterior and posterior injections are most frequently used [16], without a clear consensus in the literature on which approach is superior. Harley et al. [17,18] showed higher accuracy using the anterior approach (80%) compared to the posterior approach (50%), suggesting potentially greater reliability of the anterior injection. The introduction of ultrasound to guide injections has represented a significant advancement in clinical practice, with a substantial increase in its use over the past decade [17]. Compared with standard techniques, ultrasound guidance significantly improves procedural accuracy and reduces the risk of injury to vascular and soft tissue structures. It allows the operator to directly monitor needle placement and, in some cases, to visualize the distribution of the injected agent [19,20,21,22]. Ultrasound guidance is particularly indicated for injections targeting the sheath of the long head of the biceps tendon. Similarly, in the case of the acromioclavicular joint, where arthritic deformities and morphological variability are frequent, ultrasound significantly improves injection accuracy [23,24].

Corticosteroids are the main agents used in intra- and periarticular injections, often combined with local anesthetics. Slow-release formulations are microcrystalline suspensions that hydrolyze locally, offering prolonged anti-inflammatory effects, and can vary in potency, duration, concentration, and side effects [18,25]. Corticosteroid injections offer temporary symptom relief in managing chronic shoulder pain and can be administered into the glenohumeral joint, subacromial space, rotator cuff tendon sheaths, or trigger points.

Evidence on their clinical effectiveness remains controversial: subacromial corticosteroid injections provide modest benefits over placebo for pain and function in rotator cuff pathologies, but no clear superiority over NSAIDs has been demonstrated [20,26,27,28]. Infiltrative treatment may also be effective due to the hydrothiolation technique, which reduces pain and inflammation through capsular distension [29]. Corticosteroids contribute to these effects by decreasing synovial vascularization, leukocyte count, and the release of inflammatory mediators [30,31]. Combining ultrasound-guided injections with targeted physiotherapy may enhance clinical outcomes in order to avoid more ivasive approaches as radiofrequency or surgery [32].

Therefore, this randomized controlled trial aimed to evaluate whether, following ultrasound-guided corticosteroid injections, a rehabilitation program based on Proprioceptive Neuromuscular Facilitation provides superior clinical outcomes compared with standardized exercise-based physiotherapy in patients with subacromial bursitis.

## 2. Materials and Methods

### 2.1. Participants

Patients affected by chronic shoulder pain associated with subacromial bursitis were recruited from the Complex Operative Unit of Physical and Rehabilitation Medicine at our institution, between January 2025 and May 2025. All participants were invited to carefully read and sign an informed consent form. The researchers ensured the protection of privacy and adherence to study procedures in compliance with the Declaration of Helsinki and relevant national and international regulations. This study was conducted in accordance with the CONSORT guidelines [33] and was approved by the Ethics Committee of the Calabria Region (protocol number: 380/2024). The CONSORT checklist and participant flow diagram are provided as Supplementary Materials. The trial was registered on ClinicalTrials.gov with the following identification number: NCT07108400.

Inclusion criteria were (a) Chronic Shoulder pain associated with subacromial bursitis; (b) Adults (age ≥ 18 years); (c) Numeric Rating Scale (NRS) score ≥ 4; (d) Body Mass Index (BMI) < 30 kg/m^2^.

The exclusion criteria were (a) Severe cognitive impairment (Mini-Mental State Examination score < 24) and/or patients unable to provide informed consent; (b) Severe cardiovascular or cerebrovascular diseases; (c) History of neurological or psychiatric disorders; (d) Active oncological diseases; (e) Rheumatoid arthritis; (f) Prior shoulder arthroplasty; (g) Severe osteoporosis; (h) Use of oral analgesics, intra-articular injections, physiotherapy, and/or physical instrumental therapies in the 15 days prior to enrolment. Only patients who signed the informed consent for personal data processing were included.

### 2.2. Intervention

At baseline (T0), the patients enrolled in the study were randomly assigned (1:1 ratio) to two different treatment groups. The first was the experimental group, in which patients underwent physiotherapy based on the PNF technique; the second was the control group, in which patients received standardized exercise-based physiotherapy.

#### 2.2.1. Ultrasound-Guided Corticosteroid Injections

**Figure 1:**
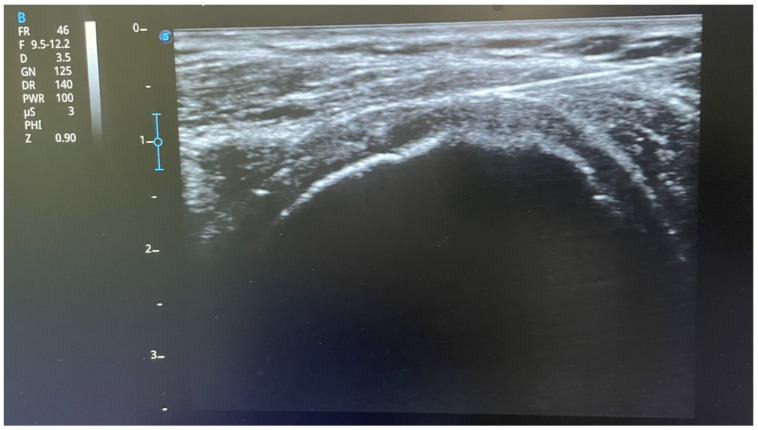
Injection at the subacromial bursa.

Before starting physiotherapy, all patients received a cycle of three once-weekly ultrasound-guided intra-bursal injections by a Physical Medicine and Rehabilitation physician. The medication used was 2 mL of triamcinolone acetonide (40 mg) with 2 mL of lidocaine and was administered using a 21-gauge needle through the anterolateral approach, with the patient in the supine position and the arm relaxed alongside the body in slight external rotation, as depicted in Figure 1.

The procedure was performed under ultrasound guidance, as it allows real-time monitoring of both needle placement and drug distribution within the bursa. This improves accuracy and reduces the likelihood of errors.

#### 2.2.2. Physiotherapy

##### Proprioceptive Neuromuscular Facilitation

The PNF intervention consisted of a 2-week treatment with five weekly hour-long sessions performed with an expert physiotherapist trained in this method. The primary goals of the program included improvement of muscle mass, improvement of strength, and improvement of muscle performance.

In detail, the 60 min rehabilitative program was as follows:

(1) At the beginning of each session, 10 min were dedicated to improving joint mobility: The facilitation technique was used at the beginning of each session to mobilize the scapulothoracic joint. The method involves mobilization of the scapular girdle along two diagonal trajectories as described by Kabat: anterior elevation, which stimulates the ipsilateral scalene muscles, and posterior depression, which activates the rhomboid muscles and the middle fibers of the trapezius [34,35].
(2) This was followed by a 15 min segment focused on improving mobility, strength, stability, and neuromuscular control, particularly in patients with motor impairments. The exercises consisted of 2–3 sets of 6–12 repetitions working at 60–80% of the patient’s one-repetition maximum weight (1RM). Specific movement patterns (such as flexion-adduction-external rotation and extension-abduction-internal rotation) were used to activate both anterior and posterior upper limb muscles. These patterns often mimicked functional tasks to encourage coordinated, multi-joint movement. To enhance effectiveness, facilitation techniques were applied: agonist reversals (combining concentric and eccentric contractions), dynamic reversals of antagonists (engaging opposing muscle groups), and rhythmic stabilization (alternating isometric contractions). This integrative approach supported not only muscle recovery but also motor coordination, timing, and proprioception, key elements in treating neurological and orthopedic conditions of the upper limb [34,35].
(3) The third part of the rehabilitation program focused on a 10 min muscle strengthening session using specific facilitation techniques focused on improving strength and motor control at an intensity from 50% to 80% 1RM, 2–3 sets of 6–12 repetitions. Agonist reversal involved alternating concentric and eccentric contractions without full muscle relaxation, especially effective in the shortened range of motion. Dynamic Reversals of Antagonists used strong contractions of opposing muscles to activate weaker agonists, guided by the therapist’s hand placements. Static Reversals, or rhythmic stabilization, relied on alternating isometric contractions to improve joint stability, particularly in the shoulder, helping the patient maintain controlled muscle activation under resistance. Together, these methods promoted both strength and neuromuscular coordination [34,35].
(4) The last part of the therapy session consisted of 10 min of stretching exercises. These maneuvers are aimed at stretching the external rotator muscles of the shoulder, the subscapularis muscle, the biceps brachii, and the pectoralis major and minor muscles.

**Figure 2:**
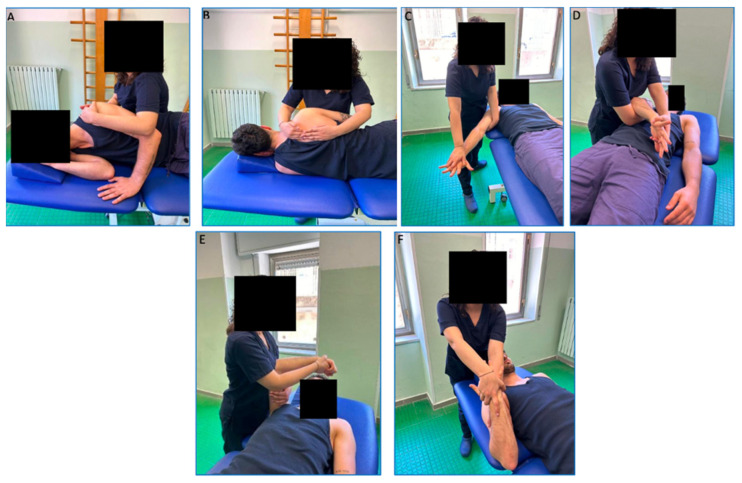
(**A**): Rhythmic initiation—anterior elevation. (**B**): Rhythmic initiation—posterior depression. (**C**): Extension, abduction, and internal rotation pattern. (**D**): Flexion, abduction, and external rotation pattern. (**E**): Flexion, abduction, and external rotation pattern. (**F**): Flexion thrust.

The rehabilitation process is shown in Figure 2.

The control group underwent a standardized exercise-based physiotherapy program lasting 45 min per session, delivered in 10 sessions over two weeks. The treatment combined passive and active kinesitherapy exercises, including isometric and isotonic contractions, alongside passive stretching performed with muscle relaxation and maintained tension for about 60 s. Passive kinesitherapy consisted of therapist-assisted shoulder mobilization. Closed kinetic chain exercises were performed in the early phase of the program, while open kinetic chain exercises were introduced progressively during treatment. Isometric exercises were performed without visible joint movement, whereas isotonic exercises involved active movement against resistance. Proprioceptive exercises were included to provide sensorimotor stimulation, and stretching exercises were performed under relaxed conditions. Joint decompression techniques were also included as part of the standardized physiotherapy protocol [34,35].

**Figure 3:**
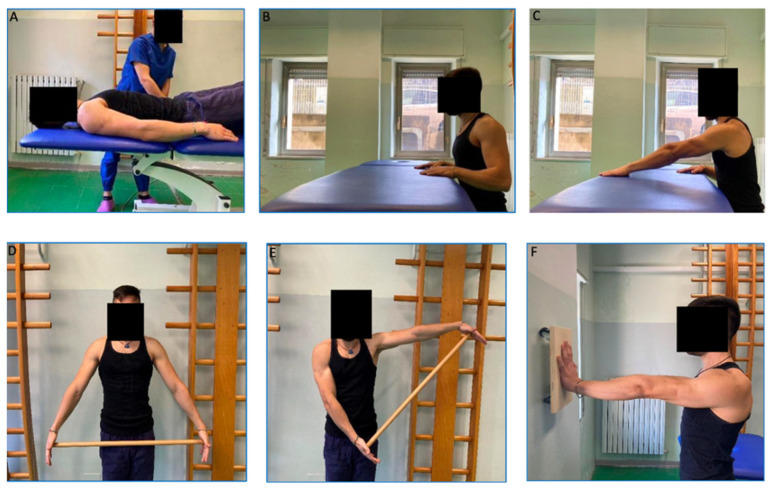
(**A**): Passive kinesitherapy: Mobilization on the sagittal plane. (**B**): Closed kinetic chain exercise: anterior elevation exercise—starting position. (**C**): Closed kinetic chain exercise; anterior elevation exercise. (**D**): Open kinetic chain exercise; abduction exercise with stick assistance. (**E**): Closed kinetic chain exercise: abduction exercise with stick assistance. (**F**): Proprioceptive exercise.

The rehabilitation process is shown in Figure 3.

### 2.3. Outcomes

Patients and the physician responsible for outcome assessments are blinded to the allocation of patients into the two study arms. The outcomes were assessed at the following time points: T0: Baseline; T1: At the end of the third injection (2 weeks from baseline); T2: At the end of the rehabilitation treatment (1 month from baseline); T3: 3 months from baseline; T4: 6 months from baseline. The secondary outcomes included the Disabilities of the Arm, Shoulder, and Hand (DASH) questionnaire, the EQ-5D, and shoulder range of motion (ROM). DASH was selected to evaluate upper-limb disability and functional impairment related to shoulder symptoms, while EQ-5D was included to assess the impact of treatment on health-related quality of life. Shoulder ROM was assessed as an additional secondary outcome to provide an objective measure of joint mobility and functional recovery following treatment.

#### 2.3.1. Numeric Rating Scale

Reduction in shoulder pain was measured using the NRS, a tool used to quantify the intensity of pain perceived by the patient during the clinical visit. The scale ranges from 0 (no pain) to 10 (worst imaginable pain). Due to its ease of use, rapid application, and ability to convert a subjective experience into a numerical value, the NRS is particularly effective for monitoring pain progression over time and evaluating the benefits of rehabilitative treatments [36].

#### 2.3.2. Disability of the Arm, Shoulder, and Hand Questionnaire

Assessment of functionality was carried out using the self-administered DASH, which consists of 38 questions divided into three sections: activities of daily living, work-related activities, and sports activities, that involve the use of the upper limb. Each question is scored from 1 (no difficulty) to 5 (unable to perform the activity). The final score is expressed as a percentage, ranging from 0% to 100%, where higher values indicate a greater degree of disability [37].

#### 2.3.3. Health-Related Quality of Life

Health-related quality of life was assessed using the EQ-5D questionnaire. The EQ-5D evaluates five dimensions of health status: mobility, self-care, usual activities, pain/discomfort, and anxiety/depression. The responses are converted into a single EQ-5D index value, which represents the patient’s overall health-related quality of life. Higher index values indicate a better health status, whereas lower values indicate poorer perceived health.

In addition, the EQ-VAS records the patient’s self-rated health on a vertical visual analogue scale ranging from 0 to 100, where 0 represents the worst imaginable health state, and 100 represents the best imaginable health state. Higher EQ-VAS scores, therefore, indicate better perceived general health [38].

#### 2.3.4. Range of Motion

Shoulder range of motion (ROM) was assessed using a standard goniometer and recorded in degrees. Active ROM was measured for flexion, abduction, internal rotation, and external rotation. Flexion and abduction were assessed in a seated or standing position, with the trunk stabilized to limit compensatory movements. Internal and external rotation were evaluated with the shoulder abducted to 90° and the elbow flexed to 90°, when tolerated. The final value was recorded at the maximum active movement achievable without excessive pain or compensatory trunk motion. Higher ROM values indicated greater shoulder mobility.

### 2.4. Statistical Analysis

Continuous variables were reported as mean ± standard deviation, while categorical variables were expressed as absolute numbers and percentages. The normality of data distribution was assessed before selecting the appropriate statistical test. Between-group comparisons at each time point were performed using independent-samples *t*-tests or Mann–Whitney U tests, as appropriate. Within-group changes over time were analyzed using repeated-measures ANOVA. Pairwise within-group comparisons between time points were performed using paired-samples *t*-tests or Wilcoxon signed-rank tests, according to data distribution. Statistical significance was set at *p* < 0.05. All statistical analyses were performed using Jamovi software version 2.3.28.

## 3. Results

**Figure 4:**
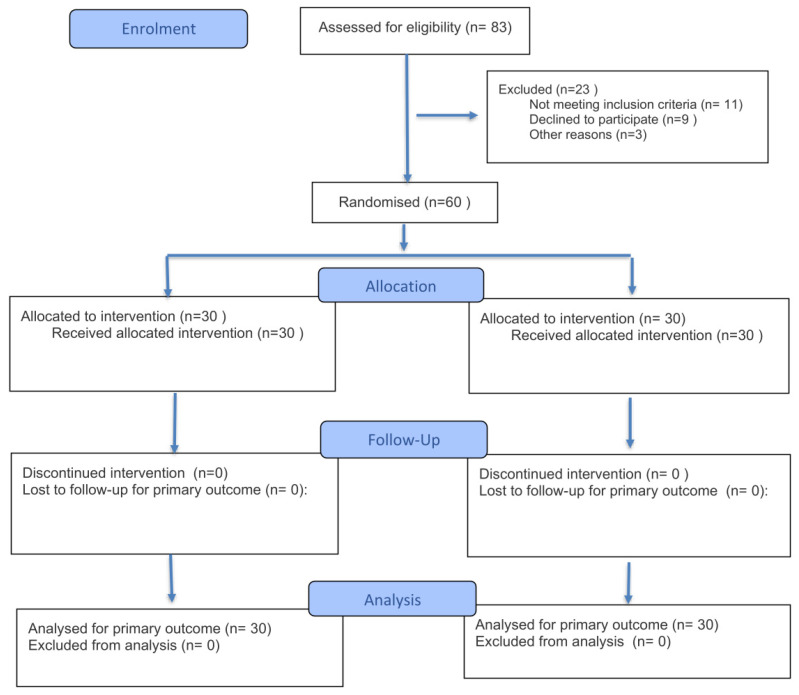
Consort flowchart diagram.

A total of 60 patients were enrolled and randomly assigned to one of the two groups, all of which completed the full protocol of three corticosteroid injections and ten rehabilitation sessions, with no major adverse events reported, as depicted in Figure 4.

The PNF group included 30 participants, 8 of whom were male (26.7%) and 22 female (73.3%), with a mean age of 64.46 ± 9.8 years. At baseline, the two groups were comparable in terms of sex distribution and BMI. However, the control group was significantly older than the PNF group (71.23 ± 10.27 vs. 64.47 ± 9.80 years; *p* = 0.011). This baseline imbalance was considered in the interpretation of the findings and acknowledged as a potential confounding factor (Table 1).

Results for primary and secondary outcomes are presented by prioritizing between-group comparisons, in accordance with the randomized controlled design of the study. Within-group changes over time are reported as complementary descriptive information to describe the evolution of each treatment arm, but they were not used as the main basis for interpreting treatment superiority.

### 3.1. Numeric Rating Scale

At baseline, no statistically significant between-group difference was observed in NRS scores (PNF: 7.33 ± 1.94; CNT: 7.47 ± 1.74; *p* = 0.780). After the injection treatment, NRS values were lower in the PNF group than in the control group, with a statistically significant between-group difference at T1 (PNF: 4.80 ± 1.92; CNT: 5.83 ± 2.00; *p* = 0.046). At the end of physiotherapy, pain scores further decreased in both groups, although the between-group difference did not reach statistical significance at T2 (PNF: 2.60 ± 2.08; CNT: 3.67 ± 2.17; *p* = 0.057). Similarly, no significant between-group difference was observed at T3 (PNF: 3.23 ± 2.50; CNT: 4.33 ± 2.45; *p* = 0.138), whereas a significant difference favoring the PNF group was detected at T4 (PNF: 3.50 ± 2.76; CNT: 4.53 ± 2.56; *p* = 0.032).

Within-group analyses showed significant reductions in NRS scores compared with baseline in both groups at all follow-up assessments. However, these changes were considered complementary descriptive findings and were not interpreted as evidence of superiority of either intervention. Detailed results of pain intensity at all time points are shown in Table 2.

### 3.2. DASH

At baseline, DASH scores were comparable between groups (PNF: 50.48 ± 12.78; CNT: 47.40 ± 16.00; *p* = 0.413). No statistically significant between-group difference was observed after the injection treatment at T1 (PNF: 34.37 ± 17.08; CNT: 49.22 ± 12.99; *p* = 0.141). After physiotherapy, DASH scores were significantly lower in the PNF group than in the control group at T2 (PNF: 14.75 ± 13.09; CNT: 26.43 ± 16.63; *p* = 0.004). This between-group difference remained significant at T3 (PNF: 20.57 ± 15.57; CNT: 29.92 ± 19.29; *p* = 0.043), but was no longer significant at T4 (PNF: 24.99 ± 19.12; CNT: 30.72 ± 20.55; *p* = 0.269).

Within-group analyses showed improvements in DASH scores over time in both groups, particularly after physiotherapy. However, these findings were interpreted as descriptive changes over time, while the main interpretation was based on between-group comparisons. The results for upper limb function are presented in Table 3.

### 3.3. EQ-VAS

At baseline, EQ-VAS scores were similar between groups (PNF: 56.83 ± 18.87; CNT: 54.00 ± 22.03; *p* = 0.595). No statistically significant between-group differences were observed at T1 (PNF: 68.33 ± 17.24; CNT: 59.83 ± 20.23; *p* = 0.085), T2 (PNF: 74.60 ± 13.56; CNT: 69.67 ± 18.89; *p* = 0.250), T3 (PNF: 75.33 ± 16.91; CNT: 69.27 ± 18.95; *p* = 0.196), or T4 (PNF: 72.17 ± 17.80; CNT: 67.67 ± 17.70; *p* = 0.330). Within-group analyses showed improvements in perceived health status over time in both groups. 

Regarding the EQ-5D-3L index, baseline values were comparable between groups (PNF: 0.60 ± 0.16; CNT: 0.62 ± 0.17; *p* = 0.651). No statistically significant between-group difference was observed at T1 (PNF: 0.74 ± 0.12; CNT: 0.72 ± 0.09; *p* = 0.419). At T2, EQ-5D-3L index values were significantly higher in the PNF group than in the control group (PNF: 0.83 ± 0.11; CNT: 0.76 ± 0.11; *p* = 0.016). Significant between-group differences were also observed at T3 (PNF: 0.85 ± 0.12; CNT: 0.78 ± 0.14; *p* = 0.033) and T4 (PNF: 0.84 ± 0.12; CNT: 0.75 ± 0.15; *p* = 0.015). Within-group analyses showed significant improvements from baseline in both groups. These findings suggest an improvement in health-related quality of life over time in both treatment arms, with between-group differences favoring the PNF group from T2 onward. Detailed results are reported in Table 3.

### 3.4. Range of Motion

At baseline, no statistically significant between-group differences were observed for shoulder flexion, abduction, extension, internal rotation, or external rotation. For flexion, between-group differences favored the PNF group from T1 onward, with significant differences at T1 (*p* = 0.003), T2 (*p* = 0.025), T3 (*p* = 0.004), and T4 (*p* = 0.009). External rotation showed a significant between-group difference only at T2. No significant between-group differences were observed for extension across the follow-up assessments.

Within-group analyses showed improvements over time in several ROM parameters in both groups. Greater ROM improvements were observed in the PNF group, particularly for flexion and abduction, with increases of +53° and +58°, respectively, compared with +40° for both movements in the control group. However, these findings should be interpreted cautiously in light of the overall study design and the common ultrasound-guided corticosteroid injection received by both groups. ROM values for flexion, abduction, internal rotation, and external rotation are reported in Table 4.

## 4. Discussion

The aim of this RCT was to compare the effectiveness of Proprioceptive Neuromuscular Facilitation and standardized exercise-based physiotherapy in patients with subacromial bursitis, after a common protocol of ultrasound-guided corticosteroid injections. Since all participants received the same injection protocol, the present study does not allow conclusions regarding the independent effectiveness of corticosteroid injections. Rather, the findings should be interpreted as reflecting the comparative effects of two rehabilitation approaches delivered after a shared infiltration protocol. Both groups showed improvements in pain, upper limb function, quality of life, and shoulder ROM over time, suggesting that both rehabilitation programs may be clinically useful when delivered after a common ultrasound-guided corticosteroid injection protocol. Although the PNF-based rehabilitation group showed greater improvements in some outcomes and at selected time points, these differences were generally modest and should not be interpreted as definitive evidence of superiority. Rather, the findings suggest a potential additional benefit of PNF-based rehabilitation compared with the standardized exercise-based physiotherapy program. Given the pilot nature of the study, the relatively small sample size, and the positive response observed in both groups, these results should be interpreted cautiously and confirmed in larger randomized controlled effectiveness studies. PNF is a technique historically used in neurological conditions; in the present study, it was associated with improvements in shoulder-related outcomes, although its specific added value over standardized exercise-based physiotherapy should be interpreted cautiously [39].

In particular, the NRS score decreased significantly over time in both groups, with a more evident reduction in the PNF group. This improvement was observed by the end of the treatment and was maintained up to six months after treatment, suggesting a possible contribution of PNF to pain control, although this interpretation should be considered cautiously given the improvements observed in both groups and the common injection protocol. This result aligns with existing literature indicating that PNF may be more effective than standardized exercise-based physiotherapy in modulating pain, likely due to proprioceptive stimulation and enhanced neuromuscular activation, which contribute to endorphin release and central pain modulation [40,41,42]. A study by Al Dajah [43] reported a significant reduction in pain using PNF combined with ultrasound therapy in patients with painful shoulders due to subacromial impingement in the short term. Our study was the first, however, to evaluate this treatment methodology in the medium-term.

In line with the randomized controlled design, the interpretation of the findings was mainly based on between-group comparisons for the predefined primary and secondary outcomes. Although both groups showed improvements over time, these intra-group changes should be interpreted with caution, as they may reflect non-specific effects, including natural recovery, regression to the mean, placebo effects, the general benefits of rehabilitation, or the effect of the ultrasound-guided corticosteroid injection administered to both groups. Therefore, the observed reduction in pain cannot be attributed solely to the specific rehabilitation program. The absence of substantial between-group differences suggests that PNF-based rehabilitation and standardized exercise-based physiotherapy may provide comparable effects after corticosteroid injection. Accordingly, the specific added value of PNF should be interpreted cautiously and requires confirmation in larger studies.

This interpretative approach was also applied to the secondary outcomes. Improvements in DASH, EQ-5D, EQ-VAS, and ROM were evaluated primarily on the basis of between-group comparisons, while within-group changes were considered descriptive. Although some secondary outcomes showed greater improvements in the PNF group at selected time points, these findings should be interpreted cautiously, particularly when between-group differences were not consistently significant across follow-up assessments. Therefore, the results suggest a possible advantage of PNF for selected functional and mobility outcomes, but they do not allow definitive conclusions regarding its superiority over standardized physiotherapy.

Numerous studies have shown that PNF stretching techniques are effective in improving joint range of motion (ROM) across various clinical contexts, surpassing standard static stretching in both short- and long-term outcomes [44,45]. These benefits are thought to stem from the neurophysiological mechanisms underlying PNF, like autogenic and reciprocal inhibition, increased stretch tolerance, and modulation of muscle tone through proprioceptive stimulation. In our study, we observed greater improvements in the PNF group compared to the standardized exercise-based physiotherapy group, particularly in shoulder flexion, extension, abduction, and rotation ROM [46,47]. These gains were maintained at three and six months from the start of treatment, although slight reductions in some of the patient-reported outcome scores suggest that booster sessions or ongoing maintenance therapy could further consolidate the improvements.

Finally, results from the DASH scale, which assesses upper limb functional disability, and the EQ-5D for quality of life, showed significant improvements at the end of the treatment, which were sustained at three and six months, with significantly greater benefit observed in the PNF group. These findings suggest a possible advantage of PNF for selected functional and quality-of-life outcomes. However, given that between-group differences were not consistently significant across all outcomes and follow-up time points, the superiority of PNF over standardized exercise-based physiotherapy cannot be definitively established.

This study has some limitations. First, the relatively small sample size limits the generalizability of the findings. Second, despite randomization, the comparison group was older than the PNF-based rehabilitation group, and age may influence pain perception, functional recovery, tissue healing, and response to rehabilitation. Third, the absence of a group not receiving ultrasound-guided corticosteroid injections prevents the independent effect of the injection protocol from being isolated. Therefore, the findings should be interpreted as reflecting the comparative effectiveness of two rehabilitation programs delivered after a common infiltration protocol, rather than the effect of corticosteroid injections alone. Other limitations include the subjective nature of pain assessment and the lack of objective biomechanical or instrumental measures, such as electromyography or motion analysis.

Nevertheless, the study has several strengths, including medium- and long-term follow-up and the inclusion of a comparison group receiving standardized exercise-based physiotherapy. In addition, excluding patients who had recently received analgesics, physiotherapy, or physical therapeutic modalities helped reduce potential confounding factors.

## 5. Conclusions

The findings suggest that both PNF-based rehabilitation and standardized exercise-based physiotherapy may improve clinical outcomes after ultrasound-guided corticosteroid injection in patients with subacromial bursitis. However, when considering the inter-group comparisons, the added value of PNF appears to be limited to selected outcomes and time points, particularly some functional and ROM measures, while no consistent superiority was observed across all predefined outcomes. Therefore, the specific superiority of PNF over standardized exercise-based physiotherapy cannot be definitively established. Larger randomized controlled trials are needed to confirm these preliminary findings and to clarify the independent contribution of the rehabilitation approach beyond the effect of corticosteroid injection.

## Figures and Tables

**Table 1:** Baseline characteristics of the sample.

	PNF Group (n = 30)	CNT Group (n = 30)	*p*-Value
Male/Female	8/22 (26.7/73.3)	8/22 (26.7/73.3)	1.000
Age	64.46 ± 9.8	71.23 ± 10.27	0.011
BMI (kg/m^2^)	26.07 ± 4.22	27.27 ± 3.44	0.230

Baseline demographic characteristics of the study population. Data are reported as mean ± standard deviation or number and percentage, as appropriate. Between-group comparisons were performed using independent-samples *t*-tests for continuous variables and Fisher’s exact test for categorical variables. PNF: Proprioceptive Neuromuscular Facilitation; CNT: Control; BMI: Body Mass Index.

**Table 2:** Intra-group and between-group differences in terms of primary outcome (Numeric Rating Scale).

		T0	T1	ΔT0–T1 *p*-Value	T2	ΔT1–T2 *p*-Value	ΔT0–T2 *p*-Value	T3	ΔT2–T3 *p*-Value	ΔT0–T3 *p*-Value	T4	ΔT3–T4 *p*-Value	ΔT0–T4 *p*-Value	Repeated Measures ANOVA
**N** **R** **S**	***Group CNT***	7.47 ± 1.74	5.83 ± 2.00	<0.001	3.67 ± 2.17	<0.001	<0.001	4.33 ± 2.45	0.083	<0.001	4.53 ± 2.56	0.511	<0.001	
	***Group PNF***	7.33 ± 1.94	4.8 ± 1.92	<0.001	2.6 ± 2.08	<0.001	<0.001	3.23 ± 2.5	0.131	<0.001	3.5 ± 2.76	0.428	<0.001	
	***Between-group p-value***	0.780	0.046		0.057			0.091			0.138			0.032

PNF: Proprioceptive Neuromuscular Facilitation; CNT: Control; T: Time points. We used independent samples *t*-test (Mann–Whitney U) and Repeated Measures ANOVA.

**Table 3:** Intra-group and between-group differences in terms of secondary outcomes (functioning and quality of life).

		T0	T1	ΔT0–T1 *p*-Value	T2	ΔT1–T2 *p*-Value	ΔT0–T2 *p*-Value	T3	ΔT2–T3 *p*-Value	ΔT0–T3 *p*-Value	T4	ΔT3–T4 *p*-Value	ΔT0–T4 *p*-Value	Repeated Measures ANOVA
**D** **A** **S** **H**	***Group CNT***	47.4 ± 16	49.22 ± 12.99	0.001	26.43 ± 16.63	<0.001	<0.001	29.92 ± 19.29	0.079	<0.001	30.72 ± 20.55	0.308	0.001	
	***Group PNF***	50.48 ± 12.78	34.37 ± 17.08	<0.001	14.75 ± 13.09	<0.001	<0.001	20.57 ± 15.57	0.040	0.008	24.99 ± 19.12	0.025	<0.001	
	***Between-group p-value***	0.413	0.141		0.004			0.043			0.269			0.099
**E** **Q** **5** **D** **3** **L**	***Group CNT***	0.62 ± 0.173	0.72 ± 0.09	0.001	0.76 ± 0.11	0.05	<0.001	0.78 ± 0.14	0.574	<0.001	0.75 ± 0.15	0.285	0.002	
	***Group PNF***	0.6 ± 0.16	0.74 ± 0.12	<0.001	0.83 ± 0.11	<0.001	<0.001	0.85 ± 0.12	0.427	<0.001	0.84 ± 0.12	0.438	<0.001	
	***Between-group p-value***	0.651	0.419		0.016			0.033			0.015			0.044
**E** **Q** **V** **A** **S**	***Group CNT***	54 ± 22.03	59.83 ± 20.23	0.04	69.67 ± 18.89	<0.001	<0.001	69.27 ± 18.95	0.892	0.001	67.67 ± 17.7	0.561	0.001	
	***Group PNF***	56.83 ± 18.87	68.33 ± 17.24	0.003	74.6 ± 13.56	0.014	<0.001	75.33 ± 16.91	0.805	<0.001	72.17 ± 17.8	0.083	<0.001	
	***Between-group p-value***	0.595	0.085		0.250			0.196			0.330			0.165

Continuous variables and parametric data are expressed as mean ± standard deviation. DASH: Disability of the Arm, Shoulder and Hand; EQ-5D-3L: EuroQol 5 Dimensions 3 Levels; EQ-VAS: EuroQol Visual Analogue Scale; CNT: Control; PNF: Proprioceptive Neuromuscular Facilitation. We used independent samples *t*-test (Mann–Whitney U) and Repeated Measures ANOVA for the outcomes of the Control and Study groups.

**Table 4:** Intra-group and between-group differences in terms of secondary outcomes (ranges of motion).

		T0	T1	ΔT0–T1 *p*-Value	T2	ΔT1–T2 *p*-Value	ΔT0–T2 *p*-Value	T3	ΔT2–T3 *p*-Value	ΔT0–T3 *p*-Value	T4	ΔT3–T4 *p*-Value	ΔT0–T4 *p*-Value	Repeated Measures ANOVA
ROM EXTENSION	***Group CNT***	34.33 ± 8.98	36.5 ± 8.82	0.030	43.3 ± 2.4	<0.001	<0.001	41.5 ± 4.94	0.032	<0.001	42 ± 4.84	0.415	<0.001	
	***Group PNF***	36.67 ± 8.02	39.33 ± 7.63	0.092	44 ± 2.42	0.002	<0.001	42.33 ± 5.37	0.106	<0.001	41.83 ± 5.49	0.083	0.001	
	***Betweengroup p-value***	0.293	0.188		0.288			0.534			0.901			0.256
ROM FLEXION	***Group CNT***	109.33 ± 42.18	122.67 ± 40.51	<0.001	148 ± 32.92	<0.001	<0.001	138.5 ± 33.86	0.002	<0.001	134.67 ± 36.46	0.089	0.0026	
	***Group PNF***	122.5 ± 28	148.67 ± 23	<0.001	163.67 ± 17.27	<0.001	<0.001	160.33 ± 21.09	0.055	<0.001	156.83 ± 25.68	0.149	<0.001	
	***Betweengroup p-value***	0.160	0.003		0.025			0.004			0.009			0.005
ROM ABDUCTION	***Group CNT***	90.83 ± 36.74	99.67 ± 40.21	0.032	124.67 ± 29.09	<0.001	<0.001	114.67 ± 33.81	0.023	0.003	111 ± 38.09	0.291	0.024	
	***Group PNF***	100.5 ± 25.03	123.33 ± 27.65	<0.001	149.67 ± 28.34	<0.001	<0.001	141.33 ± 29.09	0.056	<0.001	135 ± 31.38	0.016	<0.001	
	***Betweengroup p-value***	0.239	0.010		0.001			0.002			0.010			0.01
ROM INTRAROTATION	***Group CNT***	46.33 ± 16.24	53.67 ± 14.73	<0.001	58.17 ± 11.71	0.034	<0.001	54.83 ± 14.05	0.084	0.005	55.5 ± 14.76	0.761	0.005	
	***Group PNF***	53 ± 12.64	60.33 ± 9.99	<0.001	66.33 ± 6.29	0.001	<0.001	65.5 ± 9.93	0.098	<0.001	61.33 ± 11.59	0.062	0.002	
	***Betweengroup p-value***	0.081	0.045		0.001			0.008			0.094			0.007
ROM EXTRAROTATION	***Group CNT***	52.17 ± 14.66	57.33 ± 14.72	0.008	67 ± 15.51	<0.001	<0.001	64.17 ± 18.29	0.242	0.001	59 ± 20.1	0.031	0.081	
	***Group PNF***	58.83 ± 19.06	64.83 ± 21.27	0.096	75.66 ± 13.82	<0.001	<0.001	71.33 ± 16.5	0.052	0.002	68.5 ± 18.62	0.067	<0.021	
	***Betweengroup p-value***	0.134	0.118		0.026			0.117			0.063			0.034

Continuous variables and parametric data are expressed as mean ± standard deviation. ROM: Range of Motion; CNT: Control; PNF: Proprioceptive Neuromuscular Facilitation. We used independent samples *t*-test (Mann–Whitney U) and Repeated Measures ANOVA.

## Data Availability

The data presented in this study are available on request from the corresponding author.

## Supplementary Materials

The following supporting information can be downloaded at: https://www.mdpi.com/article/10.3390/jfmk11020224/s1. Reference [48] is cited in the supplementary materials.

## Abbreviations

The following abbreviations are used in this manuscript:

PNF	Proprioceptive Neuromuscular Facilitation
NRS	Numeric Rating Scale
DASH	Disability of the Arm, Shoulder, and Hand
EQ-5D	EuroQol 5 Dimensions
EQ-5D-3L	EuroQol 5 Dimensions 3 Levels
EQ-VAS	EuroQol Visual Analogue Scale
ROM/RoM	Range of Motion
RCT	Randomized Controlled Trial
CONSORT	Consolidated Standards of Reporting Trials
BMI	Body Mass Index
1RM	One Repetition Maximum
HRQoL	Health-Related Quality of Life
